# Association of long-COVID with major adverse cardiovascular events and mortality: a real-world data cohort study

**DOI:** 10.1186/s12872-026-06026-x

**Published:** 2026-05-25

**Authors:** Yueh-Ting Tsai, Bo-Yuan Wang, Sai-Wai Ho, Shun-Fa Yang, Yu-Hsun Wang, Chao-Bin Yeh, Ying-Cheng Chen

**Affiliations:** 1 Institute of Medicine, Chung Shan Medical University, Taichung, Taiwan; 2 Department of Emergency Medicine, School of Medicine, Chung Shan Medical University, Taichung, Taiwan; 3 Department of Emergency Medicine, Chung Shan Medical University Hospital, Taichung, Taiwan; 4 Department of Medical Research, Chung Shan Medical University Hospital, Taichung, Taiwan; 5 Department of Surgery, Changhua Christian Hospital, No. 135 Nanhsiao Street, Changhua, 500 Taiwan; 6 Department of Post-Baccalaureate Medicine, College of Medicine, National Chung Hsing University, Taichung, Taiwan

**Keywords:** Long-COVID, Major adverse cardiovascular events, Mortality, Cohort study

## Abstract

**Background:**

There is a limited body of research examining the association between long COVID and major adverse cardiovascular events (MACE) as well as all-cause mortality. This study aimed to investigate the association between long COVID and both MACE and mortality.

**Methods:**

This retrospective cohort study utilized multicenter real-world data from the TriNetX research network platform, which contains electronic health records from multiple healthcare organizations. Patients aged 18 years and older who were diagnosed with COVID-19 between 2020 and 2023 were included. The exposure group comprised individuals diagnosed with long-COVID within 3 to 6 months after their initial COVID-19 diagnosis, while the comparison group included COVID-19 patients without a diagnosis of long-COVID. The primary outcomes were the risk of major adverse cardiovascular events (MACE) and all-cause mortality. Follow-up commenced 90 days after the index date and continued until the occurrence of the study outcome or the date of the last available medical record.

**Results:**

The risk of MACE was markedly higher in the long-COVID cohort compared to the non-long-COVID cohort. The overall hazard ratio (HR) for MACE was 4.48 (95% CI: 3.95–5.07). Specific conditions such as coronary artery disease and stroke exhibited particularly high HRs, at 6.48 (5.29–7.95) and 3.46 (2.96–4.04) respectively. Mortality was significantly higher in the long-COVID group, with an HR of 1.53 (1.38–1.69).

**Conclusions:**

Compared to patients without long COVID, patients with long COVID had a higher risk of developing MACE.

**Supplementary Information:**

The online version contains supplementary material available at 10.1186/s12872-026-06026-x.

## Introduction

Since the world health organization (WHO) declared coronavirus disease 2019 (COVID-19) a pandemic in March 2020, COVID-19 has become an indelible page in human history. It has changed every aspect of our daily lives. In the following years, millions of people died from COVID-19. According to data from the US centers for Disease Control and Prevention (CDC), as of July 2025, COVID-19 has contributed to 1.2 million deaths in the US alone and more than 7 million deaths worldwide [1]. As COVID-19 vaccines have become increasingly widespread and immunity has developed after recovery, the epidemic around the world has gradually come under control. Epidemic prevention measures, including quarantine, have been gradually eased. Our daily lives have returned to normal.

However, many people did not make full recovery. The sequelae of COVID-19 continue to trouble numerous patients. COVID-19 has left a series of chronic, systemic and disabling conditions such as fatigue, chest pain, breathless, anxiety, headache and depression. A novel term “long covid,” emerged to describe these symptoms in the early stage of the COVID-19 pandemic. In a systematic review and meta-analysis investigating the long-term effect of COVID-19, the five most common symptoms were fatigue (58%), headache (44%), attention disorder (27%), hair loss (25%), and dyspnea (24%) [2]. Long covid has caused a significant public health burden in post-pandemic era. Study data suggested that more than 7% of adults and 1% of children—numbering 15 to 20 million Americans—have suffered from long covid [3, 4].

Initially, the term “long covid” had a variety of definitions. Key components— including attribution to acute infection, time course, clinical features, attention to equity, and functional impairment—differed slightly among these definitions. In 2024, the National Academies of Sciences, Engineering, and Medicine (NASEM) aimed to develop a new definition of long covid that would be accurate, precise, feasible to apply, acceptable to affected parties, accessible, and understandable. Their goal was to improve understanding of the nature, scope and burden of long covid. Ultimately NASEM defined long Covid as an infection-associated chronic condition that occurs after SARS-CoV-2 infection and is present for at least 3 months as a continuous, relapsing and remitting, or progressive disease state that affects one or more organ systems [5]. The World Health Organization (WHO) provided a slightly different definition, describing long covid as continuation or development of new symptoms 3 months after the initial SARS-CoV-2 infection [6].

These symptoms must last for at least 2 months with no other explanation. Among the various definitions of long covid, the development of chronic symptoms 3 months after the initial acute SARS-CoV-2 infection is a common consensus. With this definition, a substantial amount of related research has emerged. It has been found that long COVID symptoms can either persist continuously from the time of acute SARS-CoV-2 infection or have a delayed onset, appearing several weeks or months after recovery from the acute infection [5]. Regardless of health status, disability, socioeconomic status, age, sex, ethnic group or geographic location, long COVID can affect virtually everyone. A certain percentage of patients suffer from this condition. According to a previous study, 57% of COVID-19 survivors experienced one or more long COVID symptoms within six months of their diagnosis, while 36.55% of patients reported such symptoms between three and six months after being diagnosed with COVID-19. This poses a significant public health challenge [7].

The long-term effects of COVID-19 are a major concern for clinicians. Previous study has showed that hospitalization with pneumonia increases the risk of cardiovascular disease by 2.1 times within the first year and by 1.86 times in 10 years [8]. SARS-CoV-2 infection has been proven to lead to a series of immune mediated myocardial and microvascular injuries [9]. It also causes endothelial cell damage similar to that seen in the lungs. The cytokine storm induced by SARS-CoV-2 infection contributes to fibrofatty replacement of endothelial cells. Increased cardiometabolic demand can also cause myocardial injury through hypoxia and overexertion. Based on this theory, we hypothesized that SARS-CoV-2 infection wound lead to an increased risk of major cardiovascular events (MACE) and even death.

There are only a limited number of studies examining the relationship between long COVID and mortality or MACE. We aimed to address this gap by conducting a retrospective cohort study using the TriNetX healthcare database to investigate the association between long COVID and both MACE and mortality. Our goal was to determine whether long COVID truly leads to increased risks of MACE and death, which would help us understand the true impact of COVID-19. Additionally, we performed subgroup analyses to explore two key questions: Does the severity of the initial COVID-19 infection influence long COVID outcomes? And does timely administration of antiviral medication reduce the risk of adverse long COVID outcomes?

## Methods

### Data collection

This retrospective cohort study utilized electronic health records from TriNetX, encompassing data from approximately 118 million patients within the US collaborative network. De-identified data from multiple healthcare settings were analyzed, including hospitals, primary care, and specialty providers. The dataset comprised demographic information, diagnoses (International Classification of Diseases, Tenth Revision, Clinical Modification [ICD-10-CM]), medications (RxNorm; vaccines coded using the Code for Vaccine Administered [CVX]), procedures (International Classification of Diseases, Tenth Revision, Procedure Coding System [ICD-10-PCS] and Current Procedural Terminology [CPT]), and laboratory results (Logical Observation Identifier Names and Codes [LOINC]). The data extraction and outcome analyses were conducted on October 20, 2024. This study involved secondary analysis of existing de-identified data and was exempt from informed consent. The de-identification process complied with the Health Insurance Portability and Accountability Act (HIPAA) Privacy Rule, specifically Sect. 164.514(a), and was certified by a qualified expert in accordance with Sect. 164.514(b) [10]. This study was approved by the Institutional Review Board of Chung Shan Medical University Hospital (IRB No. CS2-23180).

### Study population

**Fig. 1:**
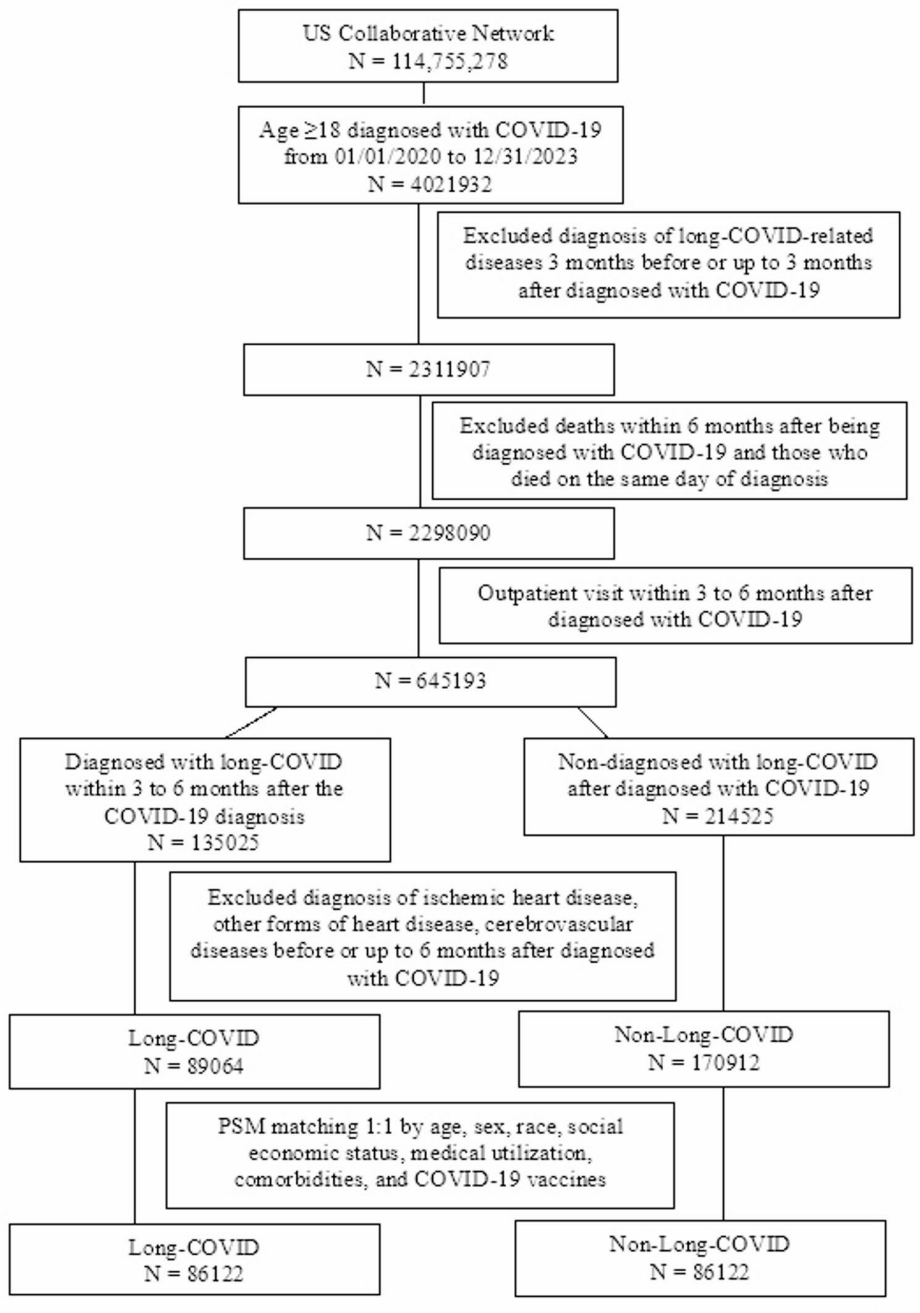
Flow-chart of subject selection

Figure 1 illustrates the flowchart detailing the construction of the study cohort. The participants included adults aged 18 years and older who had been diagnosed with COVID-19. Cases of SARS-CoV-2 infection were identified between 2020 and 2023. COVID-19 was confirmed by a positive PCR test, serologic evidence of immunoglobulins in serum or plasma, or documentation of the ICD-10-CM code U07.1 (Supplementary Table 1). Participants were excluded if they had diagnoses of long COVID–related conditions within 3 months before or after the COVID-19 diagnosis. To evaluate the robustness of the findings and reduce the potential impact of varying follow-up durations, additional analyses were conducted using fixed follow-up periods of 1 year and 3 years after the index date.

The exposure group comprised individuals who developed long COVID within 3–6 months following their initial COVID-19 diagnosis. Consistent with prior literature and established coding frameworks [7, 11], long COVID was operationally defined as post–COVID-19 conditions occurring beyond the acute phase of infection, including chest or throat pain, abnormal breathing, abdominal symptoms, fatigue, anxiety or depression, pain, headache, cognitive symptoms, and myalgia. Relevant diagnostic codes are provided in Supplementary Table 2. To reduce potential misclassification, the exposure definition incorporated both a predefined temporal window following acute COVID-19 infection and symptom-based diagnostic coding informed by previous studies.

The comparison group comprised patients who were not diagnosed with long COVID after their COVID-19 diagnosis. Both groups excluded individuals diagnosed with ischemic heart disease, other forms of heart disease, cerebrovascular diseases, pulmonary embolism, epidural hemorrhage, or subdural hemorrhage before or up to 6 months after the COVID-19 diagnosis. Baseline characteristics were gathered from medical records spanning 1 year before the index date to the day prior to it. Demographic details included age, sex, race, body mass index (BMI), and socioeconomic factors such as potential health risks related to socioeconomic and psychosocial conditions, housing and economic issues, education and literacy levels, employment status, and occupational exposure to risk factors. Medical utilization data included records of outpatient encounters, emergency department encounters, and inpatient encounters. Comorbidities were identified as nicotine dependence, alcohol-related disorders, hypertensive diseases, hyperlipidemia, chronic kidney disease, and chronic obstructive pulmonary disease, with relevant codes provided in Supplementary Table 3.

Propensity score matching was performed at a 1:1 ratio using TriNetX’s built-in function to account for baseline differences and control for confounding variables. Matching variables included age, sex, race, socioeconomic status, medical utilization, comorbidities, and COVID-19 vaccines. Propensity score matching was conducted using a greedy nearest-neighbor algorithm with a caliper width of 0.1 pooled standard deviations. The primary objective was to compare the risk of major adverse cardiovascular events (MACE, including coronary artery disease and stroke), acute myocarditis, heart failure, pulmonary embolism, and mortality (Supplementary Table 3). The observation period began 90 days after the index date and continued until the occurrence of the study outcome or the end of available medical records.

### Statistical analysis

Balance between matched cohorts was assessed using standardized mean differences (SMD), with values < 0.1 indicating adequate balance. Kaplan–Meier survival analysis and Cox proportional hazards models were used to compare risks of major adverse cardiovascular events (MACE) and mortality between the two groups. These methods provided hazard ratios (HRs) with corresponding 95% confidence intervals for robust comparisons. Stratified analyses further investigated the association between Long-COVID and MACE risk across specific subgroups. These subgroups were defined based on age, sex, race, body mass index (BMI), hospitalization status (admission within five days of COVID-19 diagnosis), and the use of antiviral medications for COVID-19. The evaluated antiviral treatments included nirmatrelvir-ritonavir, molnupiravir, remdesivir, tixagevimab, cilgavimab, and bebtelovimab, all administered within five days of diagnosis. In addition, based on the predominant circulating SARS-CoV-2 variants, we further analyzed the association between long COVID and the risk of MACE across the Alpha-dominant, Delta-dominant, and Omicron-dominant periods. All analyses were performed using the TriNetX online platform, which is powered by R version 4.0.2.

## Results

### Baseline population and clinical characteristics

The demographic and baseline characteristics of participants before and after propensity score matching (PSM) are presented in Table 1. Prior to matching, notable differences existed between the long-COVID and non-long-COVID cohorts in terms of sex, BMI, medical utility, and nicotine dependence covariates. However, after PSM, these differences were mitigated, with standardized mean differences (SMD) below 0.1, indicating effective balancing of covariates. The mean age for both groups was approximately 45 years. In terms of sex distribution, females represented 68.10% of the long-COVID group and 67.73% of the non-long-COVID group. Racial composition showed that White participants comprised approximately 72% of both cohorts, while the proportion of participants with a body mass index (BMI) of 30 or higher was similar in both groups, accounting for 28.80% and 28.87%, respectively, after matching. The mean follow-up duration was 3.42 years (standard deviation [SD]: 1.30) in the long COVID group and 2.80 years (SD: 1.46) in the non–long COVID group.

**Table 1:** Demographic characteristics of Long-COVID and non-Long-COVID

	Before PSM			After PSM		
	Long-COVID *N* = 89,064	Non-Long-COVID *N* = 170,921	SMD	Long-COVID *N* = 86,122	Non-Long-COVID *N* = 86,122	SMD
Age at Index	45.79 ± 16.44	45.81 ± 17.05	0.001	45.81 ± 16.45	45.81 ± 16.70	0.000
Sex						
Female	61,229 (68.75)	100,223 (58.64)	0.211	58,648 (68.10)	58,329 (67.73)	0.008
Male	27,816 (31.23)	70,627 (41.32)	0.211	27,455 (31.88)	27,773 (32.25)	0.008
Unknown Gender	19 (0.02)	71 (0.04)	0.011	19 (0.02)	20 (0.02)	0.001
Ethnicity						
Not Hispanic or Latino	65,039 (73.03)	120,372 (70.43)	0.058	62,766 (72.88)	63,704 (73.97)	0.025
Hispanic or Latino	7700 (8.65)	15,843 (9.27)	0.022	7479 (8.68)	7003 (8.13)	0.020
Unknown Ethnicity	16,325 (18.33)	34,706 (20.31)	0.050	15,877 (18.44)	15,415 (17.90)	0.014
Race						
White	63,566 (71.37)	111,391 (65.17)	0.134	61,351 (71.24)	62,266 (72.30)	0.024
Black or African American	13,162 (14.78)	24,518 (14.35)	0.012	12,557 (14.58)	12,622 (14.66)	0.002
Asian	3608 (4.05)	11,032 (6.45)	0.108	3601 (4.18)	3574 (4.15)	0.002
American Indian or Alaska Native	476 (0.53)	824 (0.48)	0.007	455 (0.53)	451 (0.52)	0.001
Native Hawaiian or Other Pacific Islander	624 (0.70)	1356 (0.79)	0.011	610 (0.71)	552 (0.64)	0.008
Other Race	3422 (3.84)	8743 (5.12)	0.062	3366 (3.91)	2949 (3.42)	0.026
Unknown Race	4206 (4.72)	13,057 (7.64)	0.121	4182 (4.86)	3708 (4.31)	0.026
Social economic status						
Persons with potential health hazards related to socioeconomic and psychosocial circumstances	878 (0.99)	935 (0.55)	0.050	741 (0.86)	687 (0.80)	0.007
Problems related to housing and economic circumstances	195 (0.22)	171 (0.10)	0.030	149 (0.17)	141 (0.16)	0.002
Problems related to education and literacy	18 (0.02)	23 (0.01)	0.005	16 (0.02)	18 (0.02)	0.002
Problems related to employment and unemployment	118 (0.13)	103 (0.06)	0.023	94 (0.11)	85 (0.10)	0.003
Occupational exposure to risk factors	35 (0.04)	46 (0.03)	0.007	32 (0.04)	28 (0.03)	0.002
BMI (kg/m^2^)	30.77 ± 7.69	29.48 ± 6.97	0.176	30.59 ± 7.63	30.24 ± 7.29	0.046
<30	29,712 (33.36)	50,636 (29.63)	0.080	28,665 (33.28)	29,086 (33.77)	0.010
≥30	26,985 (30.30)	33,985 (19.88)	0.242	24,803 (28.80)	24,861 (28.87)	0.001
Medical utility						
Ambulatory	73,790 (82.85)	127,668 (74.69)	0.200	70,927 (82.36)	70,636 (82.02)	0.009
Emergency	13,025 (14.62)	12,443 (7.28)	0.237	10,686 (12.41)	10,535 (12.23)	0.005
Inpatient Encounter	7823 (8.78)	10,745 (6.29)	0.095	7162 (8.32)	6967 (8.09)	0.008
Comorbidities						
Nicotine dependence	3549 (3.99)	2814 (1.65)	0.142	2512 (2.92)	2476 (2.88)	0.002
Alcohol related disorders	729 (0.82)	589 (0.35)	0.062	533 (0.62)	508 (0.59)	0.004
Hypertensive diseases	17,913 (20.11)	26,698 (15.62)	0.117	16,762 (19.46)	16,210 (18.82)	0.016
Hyperlipidemia, unspecified	8717 (9.79)	12,644 (7.40)	0.085	8142 (9.45)	7931 (9.21)	0.008
Chronic kidney disease	1622 (1.82)	3145 (1.84)	0.001	1564 (1.82)	1394 (1.62)	0.015
Other chronic obstructive pulmonary disease	1019 (1.14)	715 (0.42)	0.083	662 (0.77)	649 (0.75)	0.002
COVID-19 vaccines						
SARS-CoV-2 (COVID-19) Vaccine	14,383 (16.15)	22,216 (13.00)	0.089	13,677 (15.88)	13,243 (15.38)	0.014
COVID-19, mRNA, LNP-S, PF, 30 mcg/0.3 mL dose	1205 (1.35)	2088 (1.22)	0.012	1177 (1.37)	1132 (1.31)	0.005
COVID-19, mRNA, LNP-S, PF, 100 mcg/0.5mL dose or 50 mcg/0.25mL dose	950 (1.07)	1546 (0.91)	0.016	926 (1.08)	832 (0.97)	0.011
Severe acute respiratory syndrome coronavirus 2 (SARS-CoV-2) (coronavirus disease [COVID-19]) vaccine, mRNA-LNP, spike protein, preservative free, 30 mcg/0.3 mL dosage, diluent reconstituted, for intramuscular use	2436 (2.74)	3922 (2.30)	0.028	2295 (2.67)	2130 (2.47)	0.012
Severe acute respiratory syndrome coronavirus 2 (SARS-CoV-2) (coronavirus disease [COVID-19]) vaccine, mRNA-LNP, spike protein, preservative free, 100 mcg/0.5 mL dosage, for intramuscular use	637 (0.72)	910 (0.53)	0.023	587 (0.68)	519 (0.60)	0.010
Severe acute respiratory syndrome coronavirus 2 (SARS-CoV-2) (coronavirus disease [COVID-19]) vaccine, mRNA-LNP, spike protein, preservative free, 50 mcg/0.25 mL dosage, for intramuscular use	390 (0.44)	468 (0.27)	0.028	357 (0.42)	344 (0.40)	0.002
Severe acute respiratory syndrome coronavirus 2 (SARS-CoV-2) (coronavirus disease [COVID-19]) vaccine, mRNA-LNP, spike protein, preservative free, 30 mcg/0.3 mL dosage, tris-sucrose formulation, for intramuscular use	311 (0.35)	445 (0.26)	0.016	290 (0.34)	275 (0.32)	0.003
Severe acute respiratory syndrome coronavirus 2 (SARS-CoV-2) (coronavirus disease [COVID-19]) vaccine, DNA, spike protein, adenovirus type 26 (Ad26) vector, preservative free, 5 × 1010 viral particles/0.5 mL dosage, for intramuscular use	75 (0.08)	108 (0.06)	0.008	71 (0.08)	61 (0.07)	0.004

*SMD* Standardized mean difference

### Association on MACE and mortality

**Fig. 2:**
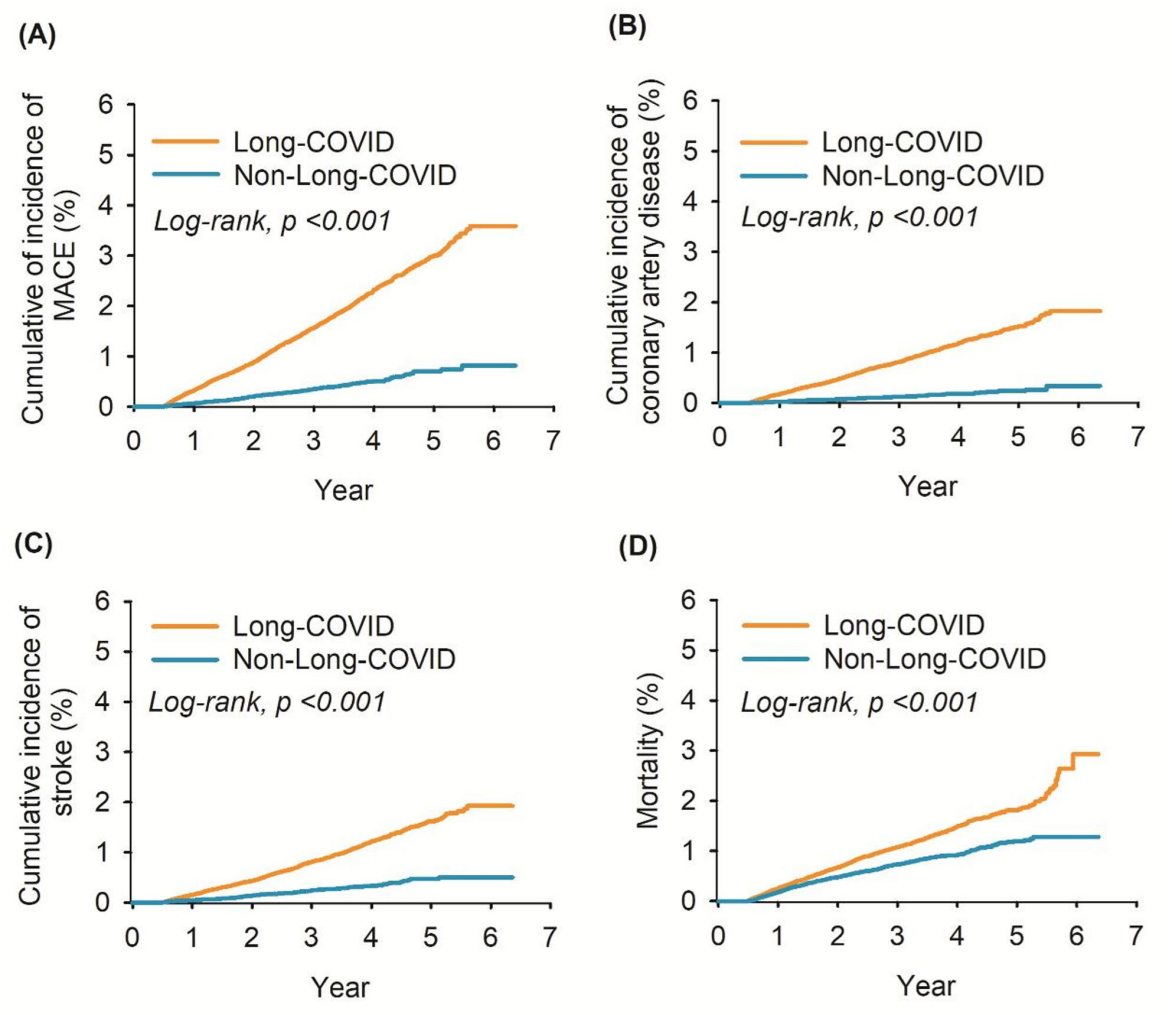
Kaplan-Meier analysis for risk of MACE. **A** MACE. **B** Coronary artery disease. **C** Stroke. **D** Mortality

The risk of MACE was markedly higher in the long-COVID cohort compared to the non-long-COVID cohort, as detailed in Table 2. Patients with long-COVID experienced an elevated risk across several cardiovascular conditions. The overall hazard ratio (HR) for MACE was 4.48 (95% CI: 3.39–5.07). Specific conditions such as coronary artery disease and stroke exhibited particularly high HRs, at 6.48 (95% CI: 5.29–7.95) and 3.46 (95% CI: 2.96–4.04), respectively. Pulmonary embolism also showed a significant increase in risk, with an HR of 4.35 (95% CI: 3.47–5.44). Mortality was significantly higher in the long-COVID group, with an HR of 1.53 (95% CI:1.38–1.69). In addition, further analyses using 1-year and 3-year follow-up periods were conducted, and the results showed a consistent trend. The Kaplan-Meier analysis, illustrated in Fig. 2, further demonstrates the increased cumulative risk of MACE over time in the long-COVID group, with event curves diverging sharply from those of the non-long-COVID group.

**Table 2:** Risk of MACE exposed to Long-COVID compared to non-Long-COVID

	No. of event			Cumulative incidence (%)		*p* for proportional hazards assumption
	Long-COVID (*N* = 86122)	Non-Long-COVID (*N* = 86122)	HR (95% CI)	Long-COVID (*N* = 86122)	Non-Long-COVID (*N* = 86122)	
Major adverse cardiovascular event	1644	291	4.48 (3.95–5.07)	3.59	0.82	0.704
Coronary artery disease	850	104	6.48 (5.29–7.95)	1.82	0.34	0.661
Acute myocardial infarction	715	95	5.94 (4.80–7.36)	1.57	0.31	0.789
Subsequent ST elevation	N/A	N/A	N/A	N/A	N/A	N/A
Certain current complications	N/A	N/A	N/A	N/A	N/A	N/A
Other acute ischemic heart diseases	210	18	9.26 (5.72–14.98)	0.43	0.04	0.468
Stroke	859	195	3.46 (2.96–4.04)	1.93	0.50	0.613
Hemorrhagic stroke	210	42	3.94 (2.83–5.49)	0.47	0.10	0.779
Cerebral infarction	711	159	3.49 (2.94–4.15)	1.63	0.43	0.389
Acute myocarditis	N/A	N/A	N/A	N/A	N/A	N/A
Heart failure	1363	244	4.37 (3.82–5.01)	4.32	0.83	0.107
Pulmonary embolism	495	90	4.35 (3.47–5.44)	1.04	0.27	0.076
Mortality	1082	566	1.53 (1.38–1.69)	2.93	1.28	0.049
Follow-up duration = 1 year						
Major adverse cardiovascular event	268	49	4.97 (3.67–6.74)	0.33	0.07	0.069
Coronary artery disease	146	19	6.98 (4.33–11.25)	0.18	0.03	0.962
Acute myocardial infarction	120	16	6.81 (4.04–11.46)	0.15	0.02	0.530
Subsequent ST elevation	N/A	N/A	N/A	N/A	N/A	N/A
Certain current complications	N/A	N/A	N/A	N/A	N/A	N/A
Other acute ischemic heart diseases	N/A	N/A	N/A	N/A	N/A	N/A
Stroke	126	33	3.47 (2.37–5.09)	0.15	0.04	0.010
Hemorrhagic stroke	N/A	N/A	N/A	N/A	N/A	N/A
Cerebral infarction	105	27	3.53 (2.32–5.39)	0.13	0.04	0.123
Acute myocarditis	N/A	N/A	N/A	N/A	N/A	N/A
Heart failure	202	41	4.47 (3.20–6.26)	0.25	0.06	0.254
Pulmonary embolism	90	11	7.44 (3.98–13.92)	0.11	0.01	0.521
Mortality	209	132	1.44 (1.15–1.79)	0.25	0.18	0.422
Follow-up duration = 3 years						
Major adverse cardiovascular event	1158	218	4.49 (3.89–5.19)	1.57	0.35	0.625
Coronary artery disease	605	77	6.65 (5.25–8.43)	0.81	0.12	0.979
Acute myocardial infarction	506	70	6.10 (4.75–7.84)	0.68	0.11	0.964
Subsequent ST elevation	N/A	N/A	N/A	N/A	N/A	N/A
Certain current complications	N/A	N/A	N/A	N/A	N/A	N/A
Other acute ischemic heart diseases	147	14	8.89 (5.14–15.37)	0.20	0.02	0.489
Stroke	594	148	3.37 (2.82–4.04)	0.81	0.24	0.977
Hemorrhagic stroke	152	29	4.41 (2.96–6.56)	0.21	0.05	0.935
Cerebral infarction	481	124	3.26 (2.67–3.97)	0.66	0.20	0.982
Acute myocarditis	N/A	N/A	N/A	N/A	N/A	N/A
Heart failure	929	170	4.61 (3.92–5.43)	1.26	0.28	0.412
Pulmonary embolism	360	64	4.74 (3.63–6.18)	0.49	0.11	0.522
Mortality	807	468	1.46 (1.31–1.64)	1.08	0.73	0.436

Based on the outcome terms, the patient count is too small, so detailed results cannot be displayed

*N/A* Not applicable

### Stratified analysis of MACE risk

**Fig. 3:**
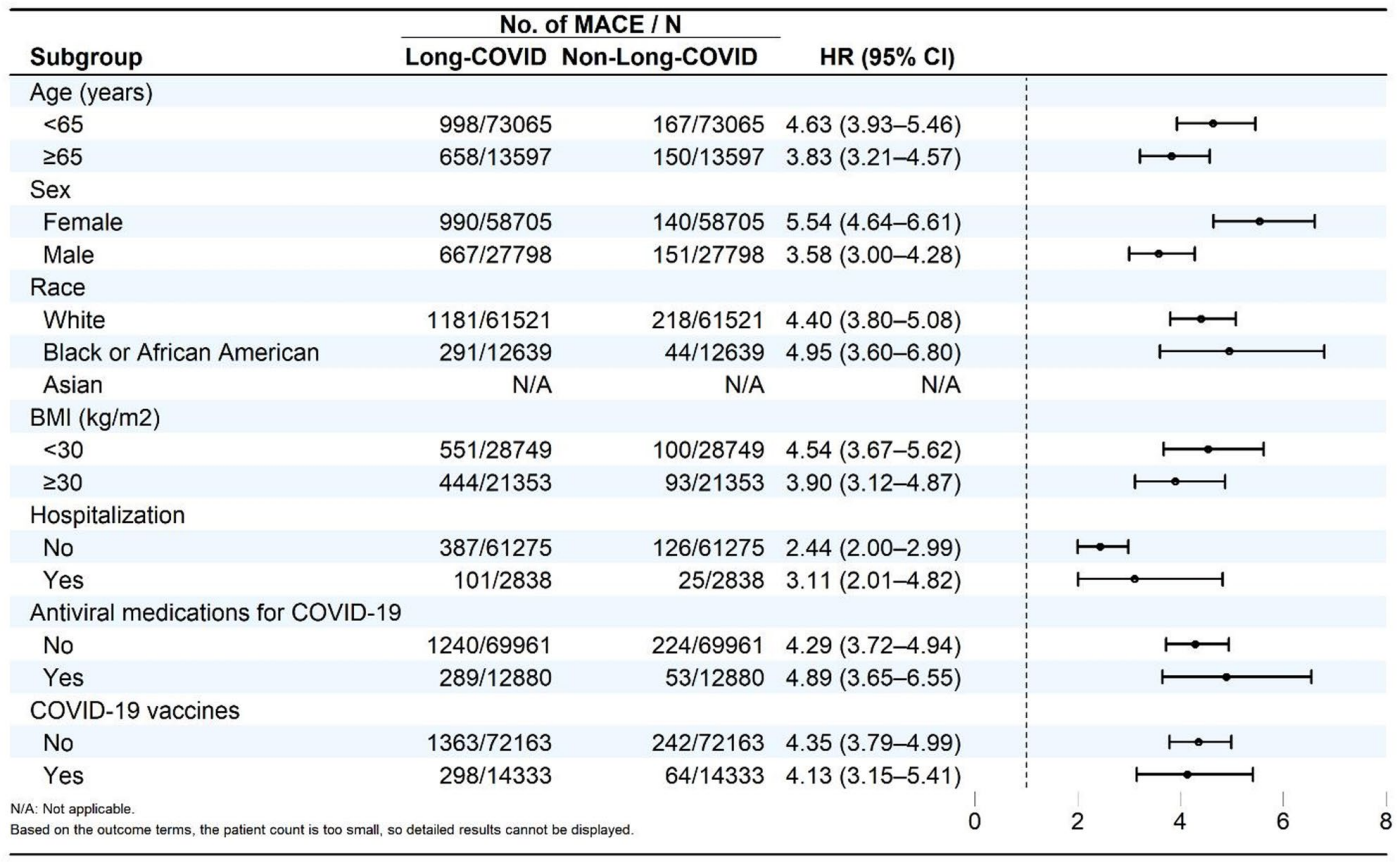
Forest-plot of stratification analysis for risk of MACE

Subgroup analyses provided further insights into risk stratification, as presented in Fig. 3. Stratified by age, sex, race, and BMI, Long-COVID demonstrated a significantly higher risk of MACE compared to Non-Long-COVID. The age-specific hazard ratios (HRs) for MACE were 4.63 (95% CI: 3.93–5.46) for participants under 65 years old and 3.83 (95% CI: 3.21–4.57) for those aged 65 and older. When stratified by sex, females had an HR of 5.54 (95% CI: 4.64–6.61), while males had an HR of 3.58 (95% CI: 3.00–4.28). Furthermore, when further categorized by hospitalization status, antiviral medication use, and COVID-19 vaccines, Long-COVID also demonstrates a significantly higher risk of MACE compared to Non-Long-COVID. Across SARS-CoV-2 variant periods, long COVID was consistently associated with a significantly higher risk of MACE compared with non–long COVID, with HRs of 4.08 (95% CI: 3.28–5.09), 4.10 (95% CI: 3.10–5.43), and 4.35 (95% CI: 3.73–5.08) during the Alpha-, Delta-, and Omicron-dominant periods, respectively (Supplementary Table 4).

## Discussion

In this study comprising 77,449 long COVID patients and a matched control group of 77,449 individuals, we observed a higher risk of major adverse cardiovascular events in long COVID patients compared to the control group. These events included acute myocardial infarction, acute ischemic heart diseases, hemorrhagic stroke, cerebral infarction, acute myocarditis, heart failure, pulmonary embolism, and subdural hemorrhage. Furthermore, we also found that long COVID patients exhibited a higher mortality rate compared to the control group (HR = 1.94, [95% CI: 1.66–2.26]). In our study, major cardiovascular adverse events—including acute myocardial infarction, stroke, heart failure, pulmonary embolism, and others— were significantly associated with long COVID. Among MACEs, long COVID patients had a higher risk of coronary artery diseases (4.82 [95% CI: 3.59–6.46]) compared to other cardiovascular complications such as acute stroke (3.67 [95% CI: 2.93–4.59]), pulmonary embolism (5.01 [95% CI: 3.66–6.87]), Heart failure (4.24 [95% CI: 3.48–5.15]).

Angiotensin-converting enzyme 2 (ACE2) plays a crucial role in the development of long COVID symptoms. SARS-CoV-2 enters human cells through the ACE2 receptor which serves as the entry point for the virus. Once inside the cell, the virus replicates and matures, triggering a series of inflammatory response [12]. Since the ACE2 receptor is found in many human organs—including the gastrointestinal tract, liver, kidneys, spleen and brain—this explains why SARS-CoV-2 can cause damage to multiple organs. The heart, in particular, expresses high levels of the ACE2 receptor, making it a significant target for SARS-CoV-2 infection [13]. Research has shown that cardiomyocytes infected by the virus undergo sarcomere disruption, fragmentation, enucleation, transcriptional changes, and a strong local immune response contributing to damage to endothelial cells and microthrombosis [14, 15]. Additionally, chronic hypoxia can increase pulmonary arterial pressure and strain on the ventricles, further worsening cardiac injury [16]. Prolonged immune activation may also cause fibrotic changes [17] and displacement of desmosomal proteins potentially leading to arrhythmias [18]. ACE2 receptors are also present in neurons meaning SARS-CoV-2 can negatively affect the autonomic nervous system. The virus-induced pro-inflammatory response can result in autonomic dysfunction and postural orthostatic tachycardia syndrome (POTS) [19–22].

Several studies have investigated the mechanisms behind COVID-19-related myocardial injury. One German study examined myocardial tissue from 39 consecutive autopsy cases who tested positive for SARS-CoV-2 via throat swab. In 16 of these patients (41%), viral loads exceeded 1000 copies per µg of RNA. A cytokine response panel consisting of 6 proinflammatory genes was increased in those 16 patients compared with 15 patients without any SARS-CoV-2 in the heart. This study demonstrates that SARS-CoV-2 can directly infect cardiomyocytes and upregulate proinflammatory gene expression, which may contribute to myocardial injury [23]. Another study assessed the relationship between SARS-CoV-2 infection and cardiac damage, finding that serum troponin T was detectable (above 3 pg/mL) in 71% of recently recovered COVID-19 patients and significantly elevated (above 13.9 pg/mL) in 5%. Furthermore, 78% of patients exhibited abnormal findings on cardiovascular magnetic resonance imaging. Endomyocardial biopsies from patients with severe abnormalities revealed active lymphocytic inflammation, confirming COVID-19-related myocardial pathology [24].

Roham et al. in 2025 reported similar findings to our study. They collected data on patients diagnosed with dementia from January 2016 to July 2023 and found a higher risk of major adverse cardiovascular events in the long COVID group compared to the control group (aHR = 1.58) [25]. However, this study defined long COVID as beginning two weeks after confirmed COVID-19 infection, which differs from the time frame used to define long COVID in our research. A retrospective study conducted across seven hospitals in Portugal analyzed 1,803 patients and revealed that 7.9% of patients hospitalized for COVID-19 experienced at least one cardiovascular event after 30 days of infection. The overall incidence rate of at least one cardiovascular event was 34.65 per 1,000 person-years (95% CI: 29.20-40.82) [26].

Another study investigating the development of new-onset cardiovascular disease within one year after COVID infection analyzed the electronic health records of 14.9 million individuals in the UK, it found a significantly increased risk of cardiovascular events, including pulmonary embolism, atrial arrhythmias, and venous thromboses, within the first four weeks post-infection (RR 6.02, 95% CI: 4.84–7.47). However, the long-term risk of cardiovascular disease was found to be lower (0.95, 95% CI: 0.85–1.06) [27]. This contrasts with the findings of our study. A possible explanation for this discrepancy could be the differing ethnicity composition of the two study populations, with differences of up to nearly 10% in certain demographic groups.

Additionally, vaccination status could also contribute to the differing results between the two studies. A 2023 systematic review and meta-analysis study found that individuals who received two vaccine doses prior to infection had a lower risk of developing long COVID symptoms compared to unvaccinated individuals [28]. Fundora et al. reviewed the protective role of COVID vaccination against cardiovascular disease (CVD). Their analysis confirmed that vaccination significantly reduces morbidity and mortality related to SARS-CoV-2 infection, including key CVD events like myocardial infarction, cerebrovascular incidents, and myopericarditis. Notably, it also mitigated risks associated with long COVID and reduced mortality in patients with pre-existing CVD [29].

COVID infection may result in long-term cardiovascular sequelae, including autonomic dysfunction, myocardial injury, left ventricular dysfunction, accelerated arteriosclerosis, and increased thrombotic risk. Therefore, cardiovascular system follow-up is essential for patients with prior COVID-19 infection, particularly among high-risk populations. A study published in 2025 utilized 24-hour Holter monitoring of heart rate variability (HRV) to evaluate whether autonomic dysfunction following acute COVID-19 infection impacted cardiovascular health. Researchers found that post-COVID patients experienced significant autonomic impairment, characterized by decreased parasympathetic activity and an elevated risk of cardiovascular complications [30]. Truong et al. performed two-dimensional speckle tracking echocardiography on 100 recovered patients with preserved left ventricular ejection fraction. Results showed a significant reduction in left ventricular global longitudinal strain in the COVID-19 group compared to controls, more pronounced in hospitalized patients. The study concluded that subclinical biventricular dysfunction may persist following COVID-19 despite preserved left ventricular ejection fraction, highlighting the importance of myocardial functional assessment in recovered COVID-19 patients [31].

The application of routine clinical biochemical assays is also beneficial in predicting cardiovascular involvement in patients with long COVID. A cohort study reported elevated high-sensitivity troponin T (hsTnT) levels and evidence of cardiac involvement detectable at 71 days post-COVID infection, even after symptom resolution [24]. A study published in 2023 explored the relationship between high sensitivity C-reactive protein (hs-CRP) and hyperglycemia with cardiovascular diseases in non-diabetic COVID ICU survivors. A one-year follow-up of 26 patients revealed elevated hs-CRP in some, with a positive correlation between hs-CRP, fasting blood sugar, and glycated hemoglobin in the high-risk group. The conclusion emphasizes the need for periodic cardiovascular check-ups for ICU survivors with elevated hs-CRP, suggesting hs-CRP as a potential early prognostic indicator [32]. Furthermore, even after recovery from COVID infection, patients who developed COVID related pulmonary fibrosis exhibit a chronic proinflammatory and prothrombotic state.

A retrospective descriptive study involving 117 patients investigated the long-term effects of COVID infection two years post-infection. Its findings revealed that patients with COVID related pulmonary fibrosis, despite showing no significant persistent dyspnea or pulmonary function impairment after recovery, had higher levels of D-dimer, mean platelet volume (MPV), lactate dehydrogenase (LDH), C-reactive protein (CRP), and neutrophils, suggesting a persistent subclinical low-grade proinflammatory and prothrombotic status [33]. From a clinical perspective, long-term cardiovascular event monitoring for long COVID patients is necessary. Studies indicate that COVID infection can lead to persistent cardiovascular sequelae, including autonomic dysfunction, myocardial injury, and subclinical cardiac dysfunction. Therefore, regular cardiovascular assessment and monitoring are crucial for long COVID patients, especially high-risk populations, to facilitate early detection and management of potential complications. Based on the current evidence, a long-term follow-up for cardiovascular events in long COVID patients is indeed clinically necessary. This follow-up not only aids in the early identification of potential complications but also provides opportunities for timely intervention, significantly improving patients’ long-term prognosis.

Furthermore, it is imperative to underscore the sustained cardiovascular burden COVID-19 imposes on individuals with pre-existing heart failure. A comprehensive 2026 systematic review characterizes COVID-19 as a chronic accelerator of cardiovascular disease within this vulnerable population. This review documented a substantial 12-month hospital readmission rate of 28% and long-term mortality rate of 18% at or beyond 12 months [34]. A profound decline in functional capacity was observed. A mean 68 m reduction in the six-minute walk distance was noted. This protracted risk driven by persistent myocardial inflammation and endothelial dysfunction uncovered the necessity of rigorous long-term surveillance. To mitigate this burden and optimize clinical outcome. post-COVID-19 management must prioritize guideline-directed medical therapy (GDMT) and the implementation of structured rehabilitation programs.

This study has several limitations. First, the study utilized data from the TriNetX database, enrolling COVID-infected patients starting from January 1, 2020. One of the inclusion criteria was the use of the ICD-10-CM code U07.1, which was officially introduced on April 1, 2020. Although this research also incorporated COVID infection determination based on serum or plasma immunoassay or respiratory specimen testing, some patients from the early pandemic period might have been missed. This could be due to variations in diagnostic standards influenced by medical resource limitations and varying healthcare environments across different regions. Second, although the TriNetX database is extensive, the distribution and data contributions from healthcare institutions within its network are uneven. Institutional heterogeneity, arising from differences in geographic distribution, size, and professional capabilities may lead to selection bias. Disproportionate data contributions from certain institutions may also potentially influence overall study outcomes. Third, COVID, as an emerging disease, experienced rapid evolution in clinical presentation, viral evolution, and therapeutic strategies. Consequently, the variability in COVID testing and case data during the pandemic presents significant limitations internationally, complicating data comparisons across different countries and time periods. Additionally, substantial differences in disease coding and diagnostic standards across various stages of the pandemic and healthcare systems might not be fully accounted for in analyses conducted using the TriNetX database. Fourth, there is a potential for residual confounding, including vaccination status and viral variants. In the present study, we have attempted to mitigate this issue by additionally adjusting for COVID-19 vaccination status in the multivariable models and incorporating variant period classification (Alpha-, Delta-, and Omicron-dominant periods) to account for temporal differences in circulating strains and treatment strategies. However, some residual confounding may still remain, as electronic health record databases often lack granular information on viral genomic sequencing at the individual level, and complete vaccination histories may be incompletely captured due to vaccinations administered outside healthcare institutions, such as community centers or pharmacies. The differential effects of vaccine type, dosing schedule, and specific viral variants on long COVID-associated MACE therefore warrant further investigation in future prospective studies. In addition, subgroup analyses should be interpreted with caution due to the lack of formal adjustment for multiple comparisons. Owing to limitations of the TriNetX platform, correction for multiple testing was not directly implemented.

Fifth, we acknowledge the potential for misclassification bias from the definition of long COVID. Long COVID is a highly heterogeneous condition lacking a single definitive diagnostic test or biomarker. Consequently, reliance on ICD-10-CM codes and symptom clusters in electronic health records may not capture all cases with perfect specificity. To mitigate this bias, we strictly adhered to standardized timeframes (3 to 6 months post-infection) and incorporated baseline medical utilization into our propensity score matching to control for healthcare-seeking behavior and detection bias.

Currently, there is no clear and standardized clinical follow-up strategy or guideline regarding the necessity of regular monitoring for cardiovascular events in patients who have recovered from COVID infection, particularly among specific populations such as older adults or those with hypertension, to facilitate early identification and intervention for related complications. With ongoing research deepening our understanding of long COVID, it is anticipated that more comprehensive knowledge will emerge, allowing for the development of more refined and targeted follow-up strategies.

## Conclusions

This multicenter real-world cohort study demonstrates that patients with long COVID have a significantly higher risk of major adverse cardiovascular events (MACE) and all-cause mortality compared with those without long COVID.

## Supplementary Information

Supplementary Material 1.

## Data Availability

Data for this study were obtained from the TriNetX network, a global federated platform providing access to de-identified electronic health record data. The underlying data are not publicly available due to data use agreements and privacy regulations.

## Abbreviations

COVID-19: Coronavirus disease 2019
ICD-10-CM: International Classification of Diseases, Tenth Revision, Clinical Modification
MACE: Major adverse cardiovascular events
PSM: Propensity score matching
SMD: Standardized mean differences
