# Supplementary material for: Association of long-COVID with major adverse cardiovascular events and mortality: a real-world data cohort study
Source: BMC Cardiovasc Disord. 2026 May 25;26:614. doi: 10.1186/s12872-026-06026-x (PMC13383476; doi:10.1186/s12872-026-06026-x)
Supplement: Supplementary file 1 — Supplementary Material 1. [file 12872_2026_6026_MOESM1_ESM.docx]

**Supplementary Table 1 Code of COVID-19**

|  | Code |
| --- | --- |
| **COVID-19** |  |
| SARS coronavirus 2 and related RNA [Presence] (labResult: Positive) | TNX: 9088 |
| SARS coronavirus 2 IgG IgM Ab [Presence] in Serum or Plasma (labResult: Positive) | TNX: 9089 |
| SARS-CoV-2 (COVID-19) IgG Ab [Units/volume] in Serum or Plasma by Immunoassay (at least 0.10 [arb'U]/mL) | LNC: 94505-5 |
| SARS-CoV-2 (COVID-19) IgM Ab [Units/volume] in Serum or Plasma by Immunoassay (at least 0.10 [arb'U]/mL) | LNC: 94506-3 |
| SARS-CoV-2 (COVID-19) IgA Ab [Presence] in Serum or Plasma by Immunoassay (labResult: Positive) | LNC: 94562-6 |
| SARS-CoV-2 (COVID-19) Ab [Presence] in Serum or Plasma by Immunoassay (labResult: Positive) | LNC: 94762-2 |
| SARS-CoV-2 (COVID-19) Ab [Units/volume] in Serum or Plasma by Immunoassay (at least 0.10 [IU]/mL) | LNC: 94769-7 |
| SARS-CoV-2 (COVID-19) Ag [Presence] in Respiratory specimen by Rapid immunoassay (labResult: Positive) | LNC: 94558-4 |
| SARS-CoV+SARS-CoV-2 (COVID-19) Ag [Presence] in Respiratory specimen by Rapid immunoassay (labResult: Positive) | LNC: 95209-3 |
| SARS-CoV-2 (COVID-19) Ag [Presence] in Upper respiratory specimen by Immunoassay (labResult: Positive) | LNC: 96119-3 |
| COVID-19 | ICD-10-CM = U07.1 |

Abbreviations: [arb'U]/mL, arbitrary unit.

LOINC, Logical Observation Identifiers Names and Codes.

ICD-10-CM: International Classification of Diseases, Tenth Revision, Clinical Modification.

**Supplementary Table 2 Code for long-COVID**

|  | ICD-10-CM |
| --- | --- |
| **Post COVID-19 condition** |  |
| Post COVID-19 condition, unspecified | U09.9 |
| Sequelae of other specified infectious and parasitic diseases | B94.8 |
| **Chest/Throat pain** |  |
| Pain in throat and chest | R07 |
| **Abnormal breathing** |  |
| Abnormalities of breathing | R06 |
| **Abdominal symptoms** |  |
| Abdominal and pelvic pain | R10 |
| Change in bowel habit | R19.4 |
| Diarrhea, unspecified | R19.7 |
| **Fatigue** |  |
| Postviral and related fatigue syndromes | G93.3 |
| Malaise and fatigue | R53 |
| **Anxiety/Depression** |  |
| Mood [affective] disorders | F30-F39 |
| Anxiety, dissociative, stress-related, somatoform and other nonpsychotic mental  disorders | F40-F48 |
| **Pain** |  |
| Pain, not elsewhere classified | G89 |
| Pain, unspecified | R52 |
| **Headache** |  |
| Headache | R51 |
| Migraine | G43 |
| Other headache syndromes | G44 |
| **Cognitive symptoms** |  |
| Vascular dementia | F01 |
| Dementia in other diseases classified elsewhere | F02 |
| Unspecified dementia | F03 |
| Delirium due to known physiological condition | F05 |
| Other specified mental disorders due to known physiological condition | F06.8 |
| Alzheimer's disease | G30 |
| Frontotemporal dementia | G31.0 |
| Neurocognitive disorder with Lewy bodies | G31.83 |
| Mild cognitive impairment of uncertain or unknown etiology | G31.84 |
| Encephalopathy, unspecified | G93.40 |
| Somnolence, stupor and coma | R40 |
| Other symptoms and signs involving cognitive functions and awareness | R41 |
| Dyslexia and other symbolic dysfunctions, not elsewhere classified | R48 |
| **Myalgia** |  |
| Myositis | M60 |
| Myalgia | M79.1 |

ICD-10-CM: International Classification of Diseases, Tenth Revision, Clinical Modification.

**Supplementary Table 3 Code for covariates and outcome**

| Content | ICD-10-CM |
| --- | --- |
| **Covariates (One year prior to the index date)** |  |
| Persons with potential health hazards related to socioeconomic and psychosocial  circumstances | Z55-Z65 |
| Problems related to housing and economic circumstances | Z59 |
| Problems related to education and literacy | Z55 |
| Problems related to employment and unemployment | Z56 |
| Occupational exposure to risk factors | Z57 |
| Nicotine dependence | F17 |
| Alcohol related disorders | F10 |
| Hypertensive diseases | I10-I1A |
| Hyperlipidemia | E78.5 |
| Chronic kidney disease | N18 |
| Chronic obstructive pulmonary disease | J44 |
| SARS-CoV-2 (COVID-19) Vaccine | CVX: 213 |
| COVID-19, mRNA, LNP-S, PF, 30 mcg/0.3 mL dose | CVX: 208 |
| COVID-19, mRNA, LNP-S, PF, 100 mcg/0.5mL dose or 50 mcg/0.25mL dose | CVX: 207 |
| Severe acute respiratory syndrome coronavirus 2 (SARS-CoV-2) (coronavirus  disease [COVID-19]) vaccine, mRNA-LNP, spike protein, preservative free, 30  mcg/0.3 mL dosage, diluent reconstituted, for intramuscular use | CPT: 91300 |
| Severe acute respiratory syndrome coronavirus 2 (SARS-CoV-2) (coronavirus  disease [COVID-19]) vaccine, mRNA-LNP, spike protein, preservative free, 100  mcg/0.5 mL dosage, for intramuscular use | CPT: 91301 |
| Severe acute respiratory syndrome coronavirus 2 (SARS-CoV-2) (coronavirus  disease [COVID-19]) vaccine, mRNA-LNP, spike protein, preservative free, 50  mcg/0.25 mL dosage, for intramuscular use | CPT: 91306 |
| Severe acute respiratory syndrome coronavirus 2 (SARS-CoV-2) (coronavirus  disease [COVID-19]) vaccine, mRNA-LNP, spike protein, preservative free, 30  mcg/0.3 mL dosage, tris-sucrose formulation, for intramuscular use | CPT: 91305 |
| Severe acute respiratory syndrome coronavirus 2 (SARS-CoV-2) (coronavirus  disease [COVID-19]) vaccine, DNA, spike protein, adenovirus type 26 (Ad26)  vector, preservative free, 5x1010 viral particles/0.5 mL dosage, for intramuscular use | CPT: 91303 |
| **Outcome** |  |
| **Major adverse cardiovascular event (MACE)** | I21-I24, I60-I63 |
| **CAD (coronary artery disease)** | I21-I24 |
| Acute myocardial infarction | I21 |
| Subsequent ST elevation (STEMI) and non-ST elevation (NSTEMI) myocardial  infarction | I22 |
| Certain current complications following ST elevation (STEMI) and non-ST  elevation (NSTEMI) myocardial infarction (within the 28 day period) | I23 |
| Other acute ischemic heart diseases | I24 |
| **Stroke** | I60-I63 |
| Hemorrhagic stroke | I60-I62 |
| Nontraumatic subarachnoid hemorrhage | I60 |
| Nontraumatic intracerebral hemorrhage | I61 |
| Other and unspecified nontraumatic intracranial hemorrhage | I62 |
| Cerebral infarction | I63 |
| **Acute myocarditis** | I40 |
| **Heart failure** | I50 |
| **Pulmonary embolism** | I26 |
| **Mortality** | - |

ICD-10-CM: International Classification of Diseases, Tenth Revision, Clinical Modification.

CVX: Vaccine administered code.

CPT: Current Procedural Terminology.

**Supplementary Table 4 Risk of MACE Across Different COVID-19 Variant Periods**

|  | No. of event | |  |
| --- | --- | --- | --- |
|  | Long-COVID | Non-Long-COVID | HR (95% CI) |
| **Alpha-dominant period** | N=23354 | N=23354 |  |
| Major adverse cardiovascular event | 512 | 94 | 4.08 (3.28–5.09) |
| Coronary artery disease | 268 | 32 | 6.25 (4.33–9.02) |
| Stroke | 277 | 65 | 3.18 (2.42–4.16) |
| Acute myocarditis | N/A | N/A | N/A |
| Heart failure | 461 | 73 | 4.67 (3.65–5.98) |
| Pulmonary embolism | 144 | 24 | 4.49 (2.92–6.92) |
| Mortality | 354 | 205 | 1.30 (1.10–1.55) |
| **Delta-dominant period** | N=17359 | N=17359 |  |
| Major adverse cardiovascular event | 308 | 58 | 4.10 (3.10–5.43) |
| Coronary artery disease | 162 | 20 | 6.26 (3.93–9.96) |
| Stroke | 157 | 38 | 3.16 (2.21–4.50) |
| Acute myocarditis | N/A | N/A | N/A |
| Heart failure | 274 | 51 | 4.15 (3.08–5.59) |
| Pulmonary embolism | 101 | 16 | 4.82 (2.84–8.17) |
| Mortality | 222 | 112 | 1.56 (1.24–1.96) |
| **Omicron-dominant period** | N=56493 | N=56493 |  |
| Major adverse cardiovascular event | 1003 | 191 | 4.35 (3.73–5.08) |
| Coronary artery disease | 514 | 73 | 5.84 (4.57–7.46) |
| Stroke | 522 | 122 | 3.51 (2.88–4.28) |
| Acute myocarditis | N/A | N/A | N/A |
| Heart failure | 755 | 157 | 3.96 (3.33–4.70) |
| Pulmonary embolism | 296 | 49 | 4.97 (3.67–6.72) |
| Mortality | 597 | 359 | 1.39 (1.22–1.59) |

N/A: Not applicable. Based on the outcome terms, the patient count is too small, so detailed results cannot be displayed.
